# Fiber Concrete Reinforced with Cord from Tires Using Low-Emission Recycling (Nearly Zero Waste)

**DOI:** 10.3390/ma19153338

**Published:** 2026-08-05

**Authors:** Andrzej Ubysz, Konrad Łuszczyk, Dominik Logoń, Aleksandra Ubysz, Jarosław Rybak

**Affiliations:** 1 Faculty of Civil Engineering, Wroclaw University of Science and Technology, 50-370 Wroclaw, Poland; 2 Faculty of Chemistry, Wroclaw University of Science and Technology, 50-370 Wroclaw, Poland; 3 Department of Civil Engineering, Wroclaw University of Environmental and Life Sciences, 50-375 Wrocław, Poland

**Keywords:** fiber concrete, tire recycling, zero waste, low-emission recycling, recycled fibers, sustainable material

## Abstract

This paper presents the possibilities of using steel cord from car tires as a structural concrete reinforcement obtained after initial mechanical treatment. The steel wire constituting tire reinforcement after mechanical separation from rubber is not further processed and, in the form partially contaminated with rubber, is stored in landfills. Further processing is usually not economically justified. The purpose of the work is to determine the physical properties of fiber concrete, made of ordinary concrete with the addition of steel fibers recovered from tires, obtained in the process of relatively easy recycling. The work is mainly experimental. The literature review presents the basics of the current state of knowledge regarding the methods of making and testing elements made of fiber-reinforced concrete. In the next part, the authors’ own research and results of testing fiber-reinforced concrete with wires after pre-cleaning are shown. Chapter five summarizes the results of research and juxtaposes the most important observations that can be used for the practical use of fibers obtained in a simple recycling technology. The utilitarian value of the work is to show the practical possibilities of recovery (recycling) of used tires, in particular for the fiber-reinforcement of concrete. Among other things, it was shown that the addition of steel fibers (contaminated with rubber) to approximately 1.3% reduces the shrinkage of concrete with these fibers by 7–10% compared to a fiber-free concrete matrix cured under the same conditions.

## 1. Introduction

Recycling waste materials has become a pressing need these days mainly due to the protection of raw materials and the protection of the environment against pollution and climate change processes [1]. Commonly used disposal methods often lead to secondary environmental pollution. For this reason, modern technologies strive to utilize waste materials as secondary raw materials to bring us closer to the idea of “zero waste” [2].

This manuscript demonstrates the potential use of recycled car tire reinforcement as a semi-finished product for the production of fiber-reinforced concrete. A distinctive feature of the proposed technology is its minimal recycling costs and energy inputs. The steel used in this technological process comes from recycled car tires.

After mechanical separation from the rubber, the steel wire constituting the carcass of these tires is not further processed, is partially contaminated with rubber, and is disposed of in landfills [3]. Further processing is usually economically unjustified. The aim of this work is to determine the physical and rheological properties of fiber-reinforced concrete—in this case, a structural material made of conventional concrete and reinforcement recovered from the direct use of rubber-contaminated steel from landfills—after simple, mechanical separation of this steel from recycled tires [4,5].

The aim of the invention is to preserve the most important mechanical properties of structural elements using 100% recycled fibers that do not require energy expenditure or produce chemical pollutants. The effectiveness of the method was confirmed by laboratory tests on full-scale elements.

The topic of using tires as recycled materials appears frequently in the scientific literature. Resource recovery from used tires was described at the end of the last century by a group of authors who presented their observations in [6,7,8]. Research results characterizing the physical properties of concrete reinforced with purified steel fibers from used car tires are presented, among others, in [9,10,11,12,13,14]. A comprehensive research program on the effect of rubber aggregates from recycled tires on the physical properties of cement concrete is presented in manuscripts [15,16]. Another group of researchers investigated the use of rubber from recycled tires as a concrete aggregate [17]. Methods for using steel cords from used tires in the design of concrete formulations were studied by, among others, Bodur [18]. Some other applications are suggested in work [19], where the authors focus on geoenvironmental utilization of waste rubber tires.

Theoretical computational models of concrete beams reinforced with mixed recycled steel fibers were presented in [20,21,22,23]. Subsequent studies addressed specific design issues, including:

- The method of fiber distribution in the tension zone [22].
- The effect of shrinkage on the strength parameters of concrete [23]; studies on the effect of rubber on foamed concrete [24]; the effect of fibers on the load-bearing capacity of a prestressed cross-section [25]; the puncture resistance of slabs reinforced with recycled fibers [26]; experimental and theoretical analysis of stress and strain distribution in fiber-reinforced bending elements [27]; the use of used tires in water systems [28].

Most of these studies addressed the broad concept of recycling and did not focus solely on car tires, which, due to their use, exhibited certain specific characteristics, such as overheating, chemical contamination, or fatigue processes [29,30].

Tire recycling did not exclusively concern reinforcing fiber cords. Another direction was replacing aggregate in concrete with crushed tires [31,32,33]. A particular area of research has focused on concrete with the addition of rubber aggregate from tires for the production of rigid pavements [34,35]. This area is attractive to designers due to its good material properties under dynamic loads. Research has been conducted on the damping properties of rubber [36] and insulating properties [37], as well as on improving the tensile strength of concrete [38,39]. In recent years, experiments have also been conducted using steel fibers from used tires to reinforce self-compacting concrete [40]. Currently, a new element in research is numerical modeling using artificial intelligence (AI), which has been applied to axially compressed columns. Both laboratory experimental studies and numerical models are primarily concerned with steel fibers subjected to physical or chemical processes involving the purification of the material introduced into the concrete formulation. A characteristic feature of these laboratory studies is, above all, low-cost recycling, allowing for the reuse of significant quantities of rubber-contaminated steel wires lying in waste dumps, whose physical and mechanical cleaning is economically unviable [41].

The authors are also familiar with doctoral dissertations concerning the use of wire derived from recycled tires as reinforcement for dispersed concrete. For example, Telemat [42] based his research on a pre-cleaned and processed fiber phase. Rubber-contaminated fibers were separated and sieved to obtain a uniform fiber structure in terms of shape and size, in accordance with the recommendations of the ASTM A-820 standard [43].

The fiber used for testing had an average diameter of 0.23 mm, a length of over 20 mm, and a tensile strength of ~2000 MPa. A significant portion of this work was devoted to fibers recovered through pyrolysis. In pyrolysis reactors, the wire is completely cleaned of rubber residues. Petroleum products are decomposed in the reactors into pyrolysis oil, pyrolysis carbon (soot), and pyrolysis gas. The process occurs at low pressure and temperatures of 350–500 °C. Chopped fibers, typically 30–40 mm long, were used for the tests. This industrial method involves significant costs for producing each cubic meter of reinforced concrete.

The research by Jafarifar [44] concerns the shrinkage behavior of fiber-reinforced concrete surfaces using steel fibers from tires, which are used for structural reinforcement. Due to this topic, a significant portion of the work was devoted to the mechanisms of moisture transport and concrete drying. As in the previously cited work, the author used highly processed wire for the tests. It contained less than 2% by mass of rubber impurities. The fiber length was 50% wires of 15–25 mm, and no more than 20% steel fibers longer than 25 mm. The monograph analyzed and investigated the deformation of fiber-reinforced slabs and the changes in the strength of the near-surface layer, where shrinkage cracks develop at a depth of approximately ¼ of the slab thickness.

Yasin [45] presented the results of his own approach to shrinkage testing on beam-shaped specimens with enlarged heads on both sides, in which bolts were embedded in concrete. The threads of the embedded bolts extended beyond the beams’ outline. Using these bolts, the beams were fixed on both sides in a rigid steel frame, where cracks and their opening in the central section were examined. Cleaned wires with lengths of 3–30 mm, with a permissible quantitative tolerance, were also used to prepare the beams made of concrete containing crushed tire aggregate. A fiber mixture was allowed in which up to 5% of the total amount could be shorter than 3 mm and 5% could be longer than 30 mm.

The authors of the above-mentioned studies compared composites reinforced with factory-made fibers to composites reinforced with recycled fibers. An analysis of the available literature clearly indicates that concretes structurally reinforced with factory-made fibers achieve higher mechanical properties than concretes reinforced with fibers derived from recycled car tires.

This topic has also been the subject of several patent applications. Among them, [46] presents a cement panel in which a reinforcing layer of basalt fibers is embedded in at least one of the main surfaces. The solution introduces meshes or fabrics made of basalt fibers (continuous, chopped, or micro-fibers) with porosity that allows the cement core material to penetrate during the embedding of the layer on one or both surfaces of the panel. This application results in easier fabrication and improved mechanical and performance properties, including tensile and flexural strength, impact strength, stiffness, temperature and alkali resistance, dimensional uniformity, surface smoothness, resistance to water penetration, and increased edge resistance to connector pullout.

The US patent application [47] concerns a method for designing a synthetic fiber mixture with varying linear density, cross-section, and slenderness, adapted to the mortar grain size in continuous aggregate concrete, in order to achieve optimal fiber distribution in the matrix and reduce crack initiation. The solution envisages a mixture content of approximately 0.025–1.0% by volume, and the method involves adding this mixture to concrete and uniformly distributing it during mixing.

This application results in improved crack resistance, reduced crack initiation, increased impact strength, and reduced construction time.

Another US application [48] concerns a method for producing glass fiber-reinforced concrete. The solution introduces strictly controlled process and composition parameters: mixing water temperature (preferably around 63–67 °F), short heating times in the range of 80–150 °F for the pre-cure and cure stages, a total pre-cure + cure time of less than 110 min, defined ranges of cement, sand, polymer, and water content and typical layer thicknesses, as well as a set of process tools enabling the shaping of internal voids and application by spraying or vibrocasting. The result is a significant reduction in production cycle time from days to hours while maintaining the required strength parameters, improved layer bonding, the ability to reduce product weight and produce thin-walled, complex shapes, as well as higher quality repeatability and simplified production.

A well-known method for manufacturing reinforced concrete elements involves preparing a mesh of threads in the first plane. Reinforcing fibers are then applied perpendicularly or diagonally to this plane, and the mesh with the applied fibers is then embedded in the cement mass (US application [49]). The solution introduces a mesh carrier for controlled fiber deposition and techniques for fixing them (including gluing, fiber jet blowing, or lifting the fibers with rollers and fixing them). This provides an open structure permeable to the grout and enables the creation of a multidirectional, or even three-dimensional, reinforcement system and local reinforcement of high-stress zones. The result is easier and faster production while simultaneously increasing and uniforming the strength of products, reducing fiber mixing problems in concrete, and improving the crack resistance and operational load resistance of slabs and precast elements.

Formulations are commonly used to reinforce concrete, distributing fibers throughout the cross-section. Numerous scientific studies and engineering practice indicate that appropriately selected fiber additions (steel, polypropylene, basalt, glass, or carbon) can increase tensile and flexural strength, limit crack propagation by bridging microcracks, reduce shrinkage cracks, and modify the concrete’s failure mode from brittle to more ductile. At the same time, increasing fiber content can lead to technological problems related to reduced mix workability, caking, and uneven dispersion throughout the cross-section.

The aim of the research was to experimentally verify the possibility of using steel fibers obtained from the recycling of car tires without incurring additional financial outlays for further processing of the wire, and the use of expensive chemical admixtures in concrete to increase its workability. Such a proposal supported by laboratory experiments will contribute to the protection of the natural environment by reducing the emission of burdensome waste. The research is also intended to show the effectiveness of uncomplicated technological processes for the practical use of waste for simple structural elements.

## 2. Materials and Methods

### 2.1. Tire Processing Technologies

Several methods are currently used to process used tires. Below are examples of some of the more popular ones.

The first method involves shredding the tires and using special devices called scrapers, which separate the product into two components: rubber with a polymer matrix and wires contaminated with rubber to varying degrees. Rubber is a valuable ingredient and is widely used as an additive to asphalt mixes, for creating subbases for polyurethane pitches, as a granulate for filling artificial turf surfaces, as an additive to lower-calorie solid fuels to improve their performance, and many other applications.

Currently, due to the rubber contamination and its low weight-to-volume ratio, wire is not melted down in steel mills and, consequently, ends up in landfills. On average, 9 kg of tires yields approximately 1 kg of wire with a diameter of 0.3–1.3 mm. Taking Europe as an example, this method is used to recycle approximately 35% of all tires. Knowing that approximately 3 million tons of tires are currently recycled in Europe each year, this translates to a use of this method of up to 1 million tons of tires. Therefore, over 100,000 tons of wire are currently sent to landfills annually.

The second method involves incinerating them entirely in specially designed technological lines. This is the most popular method, primarily used by cement plants, where the combustion residue is sent to the clinker depot.

The third method involves decomposing the rubber in special devices (pyrolysis) into pyrolysis oil, pyrolysis gas, and carbon black (elementally pure carbon with impurities), using, among other methods, the water-jet method [50,51]. This method offers significant potential, as it produces a completely rubber-free wire.

A fourth, less popular recycling method involves using whole tires to protect port quays, build playgrounds, and even reinforce river banks and embankment structures. However, this has negligible value on a national scale. Other methods are also used, with negligible use. These methods account for approximately 5% of the total annual weight of used tires [52].

### 2.2. Wire Rubber Contamination

**Figure 1:**
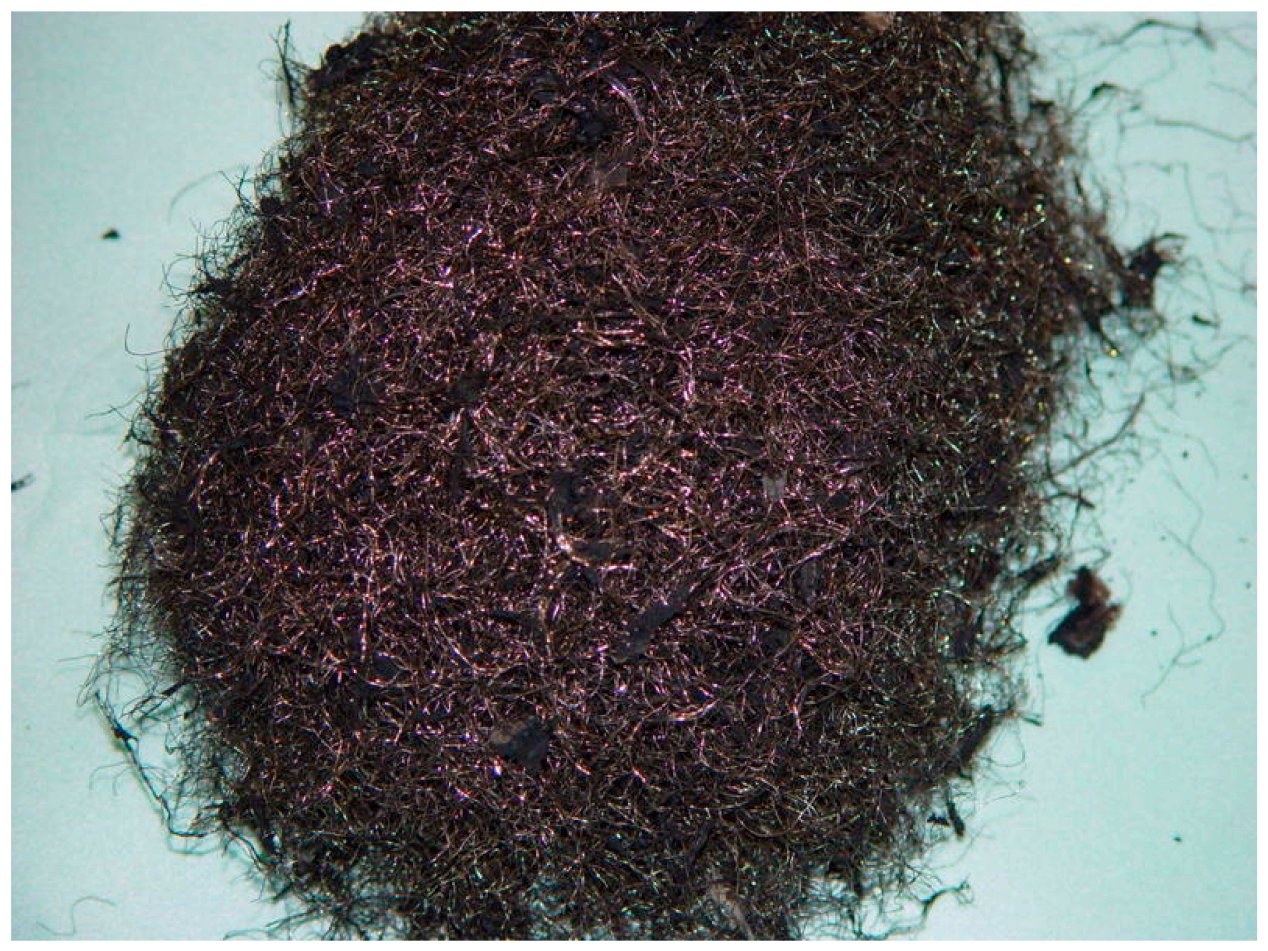
Recycled tire wire obtained from “plucking machines”.

The rubber contamination level of the wire used for the study was examined. For this purpose, 689.70 g of recycled material (as shown in Figure 1) was thoroughly cleaned, resulting in the separation of 52.05 g of rubber and nylon cord, and 637.65 g of steel wire of various thicknesses and lengths. This results in a weight contamination of ~7.56%. Considering the density of SBR rubber of ρ = 1400–1600 kg/m^3^ (an average density of ρ = 1500 kg/m^3^ was assumed for calculations), the volume contamination is ~29.78%. This means that every 1 kg of recycled fibers contains 925.9 g of steel cord and 74.1 g of rubber.

The composition of the recycled wire mixture was tested for the specific wire thicknesses. Randomly sampled wire weighing 691.24 g was separated into three fractions with thicknesses:

- ≥0.45 mm (fine wires);
- 0.46–0.95 mm (medium-thick wires);
- <0.95 mm (thick wires).

This separation resulted in the following percentage composition of the individual fractions:

- Fine wires 79.8%;
- Medium-thick wires 18.5%;
- Thick wires 1.7%.

### 2.3. Concrete Formulations

For series 1 and 1a, ready-mixed concrete of grade C20/25 was used, while for series 2, ready-mixed concrete of grade C45/55. Each series was a separate batch of concrete delivered from a concrete plant on a different date. Steel fibers obtained from recycled tires (uncleaned and unsorted) were added to the delivered concrete in the following amounts:

- For series 1—0.00%, 0.33% (25 kg/m^3^);
- For series 2—0.00%, 0.13% (10 kg/m^3^), 0.27% (20 kg/m^3^), 0.40% (30 kg/m^3^).

For series 3 and 4, the concrete was prepared in the laboratory at Wroclaw University of Science and Technology according to the following procedure:

- Gravel with a fraction of 8–16 mm—780 kg;
- Gravel with a fraction of 2–6 mm—430 kg;
- Cement CEM III/A 32.5 LH/HSR/NA—350 kg;
- River sand—700 kg;
- Water—185 L.

Tire recycling fibers were also added to the prepared concrete in the following amounts:

- For series 3—0.00%, 1.27% (95 kg/m^3^);
- For series 4—0.00%, 0.25% (19 kg/m^3^), 0.5% (38 kg/m^3^), 0.76% (57 kg/m^3^), 1.0% (76 kg/m^3^), 1.27% (95 kg/m^3^).

During concreting, the maximum amount of untreated steel tire fibers that could be extruded under laboratory conditions was determined by successively adding them to the concrete mixer while controlling the added fiber mass. For each series, samples were prepared in the following shapes:

- Cylinders ø113 mm, length l = 300 mm, with and without recycled fibers;
- Cylinders ø150 mm, length l = 700 mm, with and without recycled fibers;
- Cubes 150 × 150 × 150 mm, with and without recycled fibers;
- Beams 150 × 150 × 600 mm, with and without recycled fibers.

### 2.4. Determination of Wire Distribution in the Concrete Matrix

Fiber distribution testing was performed on samples (beams) from series 1 and 2. The beams were previously broken. After fracture, they were cut at 1/3 and 2/3 of their span—outside the fracture zone. A high-speed stationary concrete saw with water lubrication was used to cut the beams. The surfaces of the cut samples were smooth, and their edges were not frayed. Analysis of the results presented below reveals satisfactory uniformity of the wire distribution in both cut planes for each of the analyzed samples. At the same time, significant heterogeneity in the fiber distribution was observed when analyzing the results for the sample group. Although the samples were always concreted under the same conditions, in one extreme case, a coefficient of variation of up to ~61% was obtained. This significant heterogeneity is explained by the very irregular shape of the fibers (including groups of wires connected by rubber remnants) and, consequently, their tendency to curl. Hence, significantly larger clusters of wires were present in some areas than in others (Table 1).

## 3. Results of Laboratory Tests

The tests included a total of four batches of concrete with varying percentages of untreated wire, originally used in tire carcasses. The matrices used in subsequent tests had varying compressive strengths of f_ck_ ≈ 25–60 MPa (acc. [53] C20/25–C50/60). The strength of the tire carcass wire was also tested. The tests also considered the contamination level of recycled fibers.

Concrete mixtures used:

- Gravel with a fraction of 8–16 mm—780 kg;
- Gravel with a fraction of 2–6 mm—430 kg;
- Cement CEM III/A 32.5 LH/HSR/NA—350 kg;
- River sand—700 kg;
- Water—185 L.

and

- Gravel with a fraction of 8–16 mm—780 kg;
- Gravel with a fraction of 2–6 mm—430 kg;
- Cement CEM I 32.5—350 kg;
- River sand—700 kg;
- Water—185 L.

During the tests, the physical and rheological parameters of the hardened concrete were assessed, and in batches 3 and 4, the limiting value of recycled fiber addition was determined, allowing for the mixing of the mixture without the addition of superplasticizers. The uniformity of fiber distribution in the concrete matrix was also verified.

### 3.1. Wire Strength Tests

**Figure 2:**
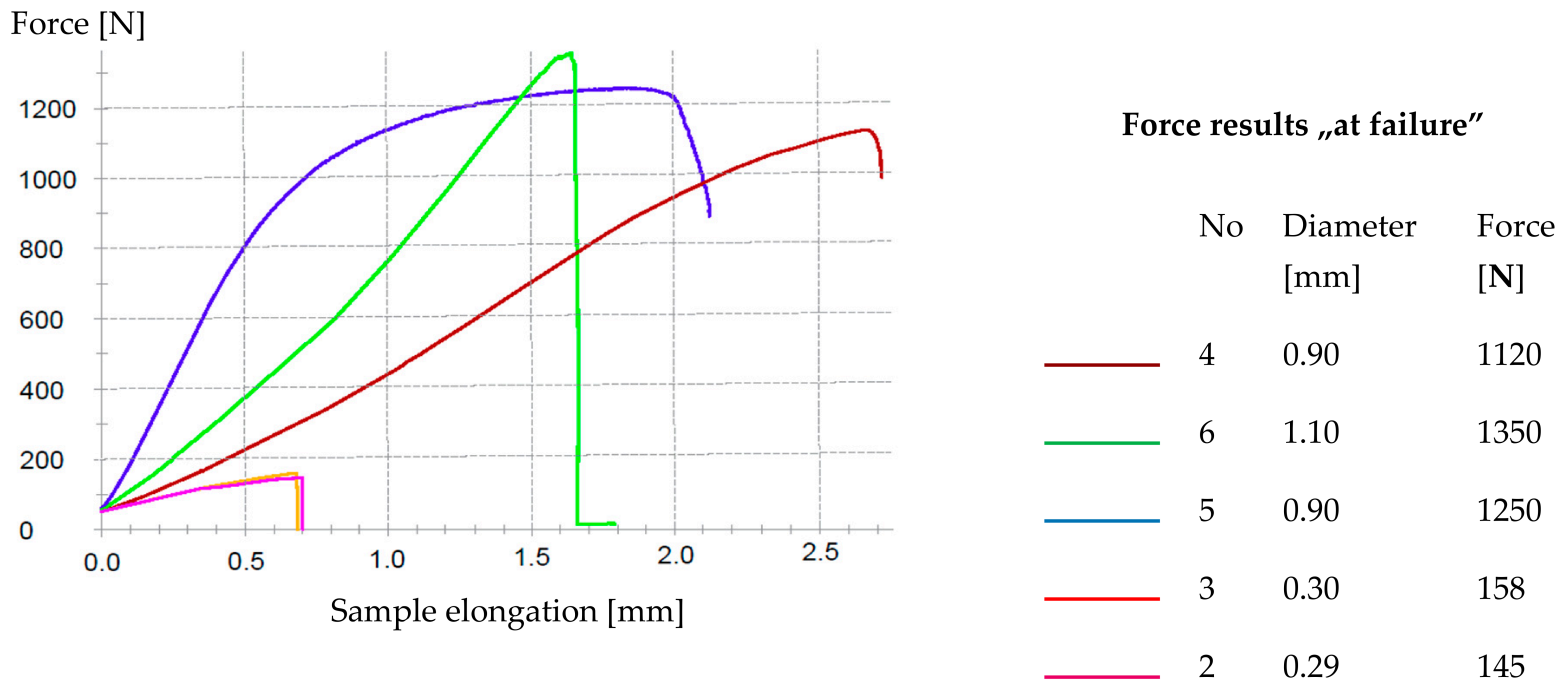
Elongation–force graphs obtained from a testing machine.

The tests were performed for various wire diameters (fibers). For 0.3 mm diameter fibers, the results were compared with the Telemat test. Average test values are presented in Table 2, and selected test graphs are presented in Figure 2.

### 3.2. Testing of Composite Strength and Stiffness in Uniaxial Compression

**Figure 3:**
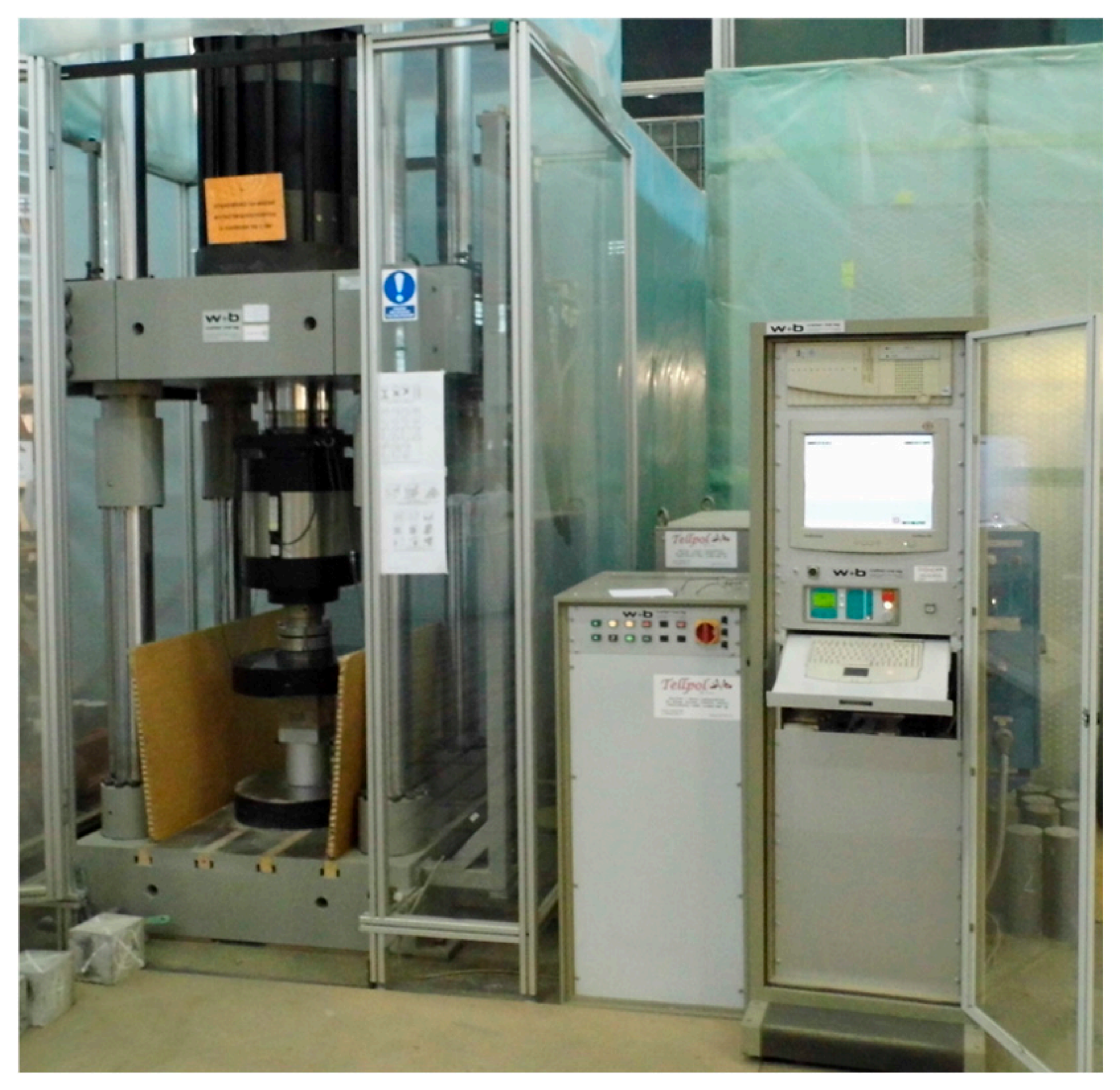
Walter+Bai a.g. strength testing machine used to test concrete compressive strength, tensile strength, and modulus of elasticity.

Before testing, the samples were stored on water grids. They were removed approximately 4 h before testing, measured, and compressed in testing machines. Concrete compressive strength tests were performed on cubes with nominal dimensions of 150 × 150 × 150 mm using Walter+Bai a.g. (Löhningen, Switzerland) (see Figure 3 below) and VEB ZD-40 testing machines (WPM Leipzig, Markkleeberg, Germany). The tests were conducted based on the guidelines provided in [54]. The samples were loaded transversely to the concreting direction with a constant stress increase within the range of 0.6 ± 0.4 MPa/s until the failure force was reached, from which the concrete compressive strength was calculated. The concrete density of the samples, mean strength, standard deviation, and coefficient of variation were also calculated. The test results are summarized in Table 3 and Table 4.

Analyzing the above-mentioned compressive strength test results for concrete, performed on 150 × 150 × 150 mm cube-shaped samples, a comparable average compressive strength was observed for the concrete compared to the matrix.

**Figure 4:**
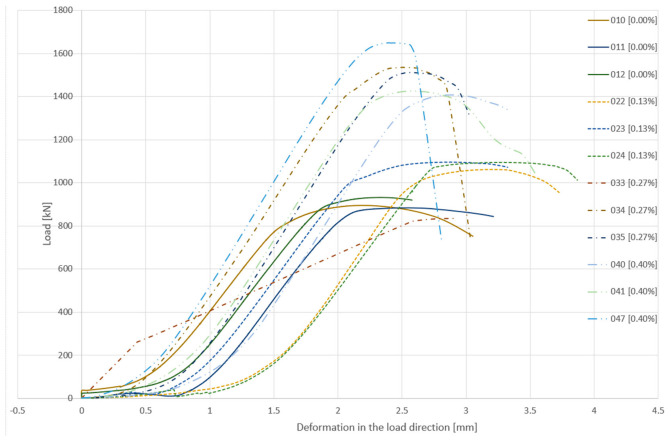
Deformation of 150 × 150 × 150 mm cube-shaped concrete specimens during compressive strength testing.

**Figure 5:**
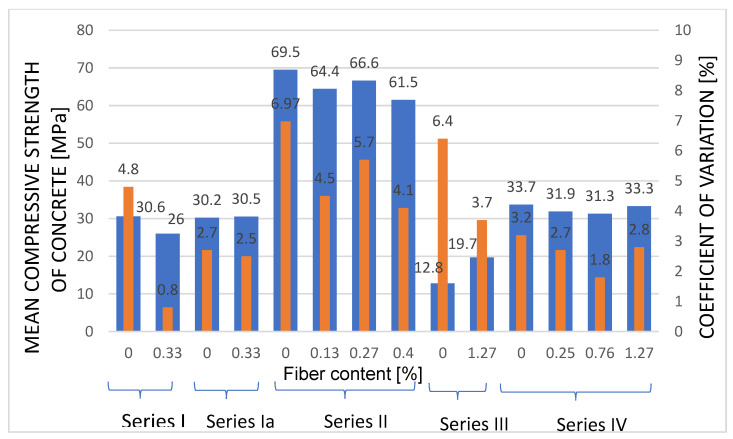
Comparison of the change in compressive strength of samples from series 1–4 (blue bars), relative to the coefficient of variation of the tested sample series (orange bars). Concrete compressive strength and the coefficient of variation.

The failure mechanism for the samples with the added fiber phase was stable—no sudden, explosive failure occurred. The strain paths of the samples were analyzed during load increments (Figure 4). Despite the introduction of heterogeneous material into the concrete, the samples achieved improved performance parameters in terms of compressive strength uniformity—this can be observed by comparing the coefficients of variation—almost every group of samples with recycled wire achieved lower coefficients of variation after reaching 28-day strength than their matrix without fibers (Figure 5).

Table 5 shows results of concrete compressive tests performed in individual series 1 and 1a. Each series is a separate batch of concrete. 

### 3.3. Concrete Resistance to Brittle Fracture

**Figure 6:**
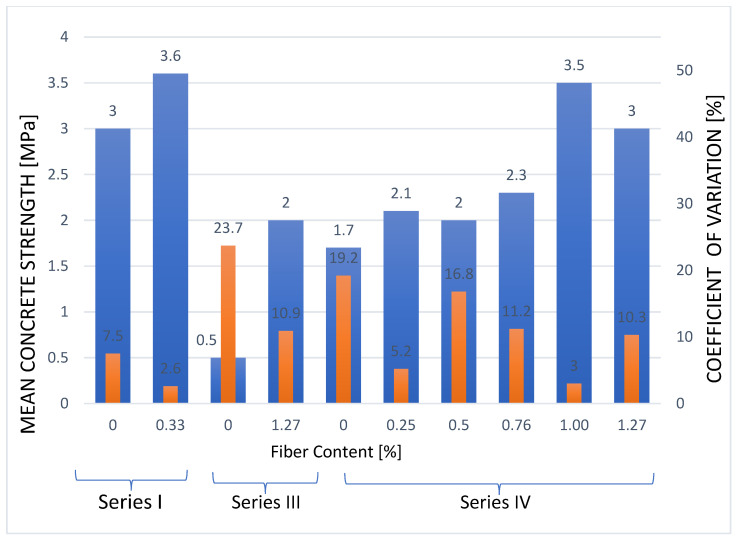
Mean concrete strength under brittle fracture in Brazilian splitting test (blue bars) and the coefficient of variation (orange bars).

Concrete resistance to brittle fracture was determined using the Brazilian method. The tests were conducted using the same testing machine as for compression. The test procedure was based on the manual [54]. Samples were removed from the water baths approximately 4 h before testing. Analyzing the results of individual test series, which were conducted to determine the variability of fiber-reinforced concrete parameters, it was observed that with increasing recycled fiber content, the most optimal concrete was with 1.00% fiber. Increase in the brittle fracture strength of samples (Brazilian method), depending on the fibrous phase content and change in the coefficient of variation is observed in Figure 6. The increase in brittle fracture resistance exceeded 200% based on average values for concrete with 1.27% fiber; the increase in strength relative to the matrix was approximately 175%. It was also observed that in the case of weak matrices, the increase in strength of concrete with the fiber phase reached significantly higher values than those described above. With 1.27% steel fiber, an average strength increase of nearly 400% was achieved. Noteworthy is the significant reduction in the coefficient of variation in samples with the addition of recycled fiber. This indicates that the composite behaved more uniformly during testing (the destructive forces of individual samples exhibited less scatter within a given series). A standardized test method was used in the study, relating the results to standard formulas. However, it should be noted that the Brazilian method is not universally accepted for determining the tensile strength of fiber-reinforced concrete—as is the case with a concrete matrix (0% fibers). The study aimed to determine the increase in brittle fracture strength of concrete reinforced with recycled steel fiber compared to a concrete matrix. This study also allows for the conclusion that this reinforcement method would significantly improve the punching shear strength of fiber-reinforced concrete (e.g., slabs). Table 6 shows results of brittle fracture resistance tests of concrete achieved in Brazilian splitting test method.

### 3.4. Concrete Flexural Tensile Strength Tests

**Figure 7:**
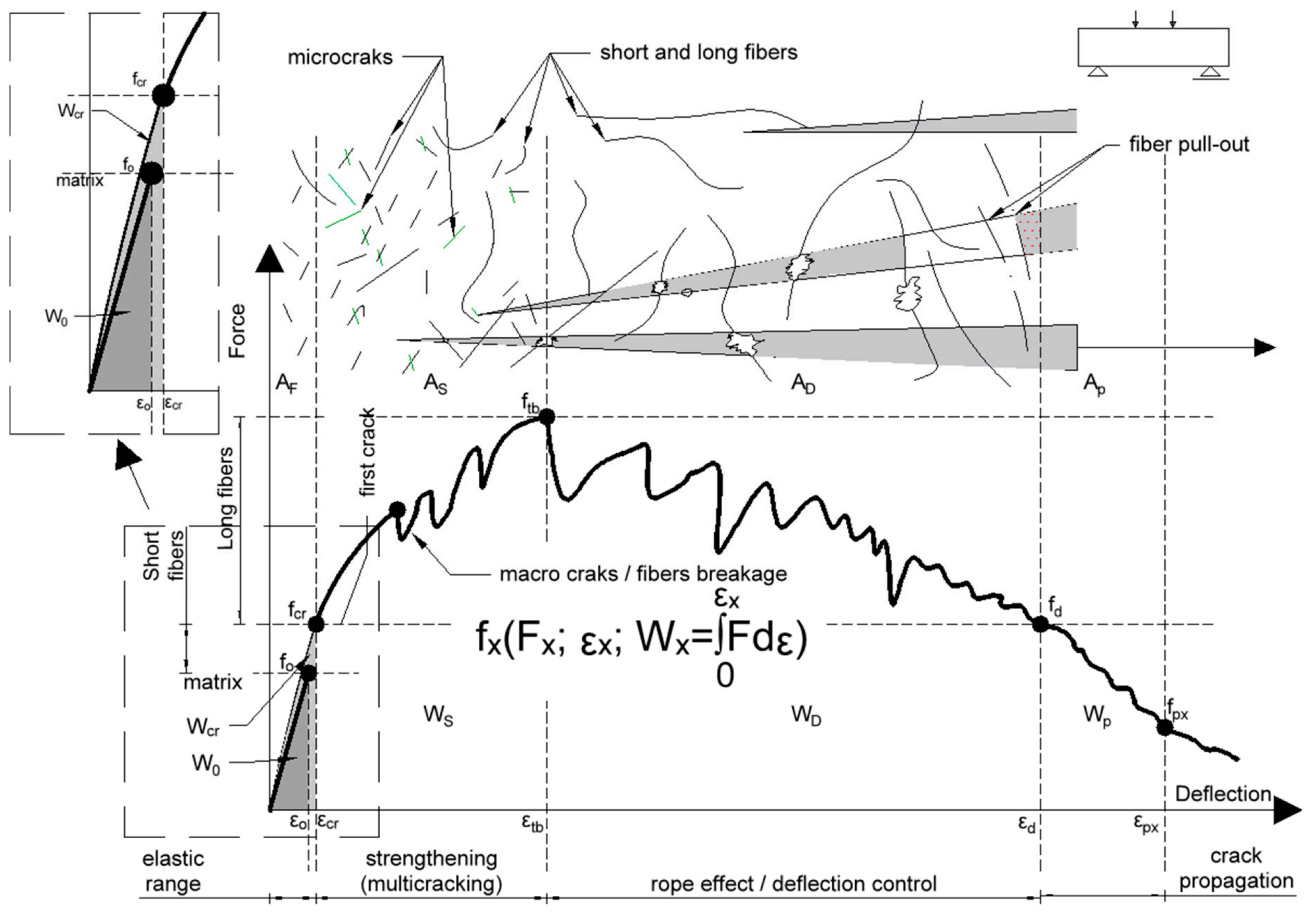
Schematic diagram of the s-e relationship in the FSD/ESD method according to Logoń [57].

Concrete flexural tensile strength tests were conducted using VEB ZD-40 testing machines on 150 × 150 × 600 mm beam samples with and without fibers. The test was based on the instructions [37]. Samples from series 1 and 2 were cured in climatic chambers at a temperature of T ≈ 21 °C ± 1 °C and air humidity of RH ≈ 55% ± 2%. The samples of series 3 and 4 were tested 36 and 30 days after concreting, respectively, while the beams of series 1 and 2 were tested at a later age (532 and 470 days, respectively; two beams of series 2—after 836 days). The analysis was performed using the multi-cracking method, which illustrates the composite strengthening phase in the range of forces from the cracking force F_cr_ to the maximum value F_tb_. This method is also known as the rope effect and the First Strengthening Deflection/Emergency Shut-down (FSD/ESD) test [55,56]. A schematic diagram of the strain–load relationship in the FSD/ESD method according to Logoń [57,58] is given in Figure 7. In the first range (Figure 7) (ε_0_-ε_cr_), the composite works elastically (proportional region), followed by a material strengthening phase (ε_cr_-ε_tb_), often called multi-cracking. In this phase, macro-cracks begin to form, and the first fibers are torn/pulled out.

Distinct undulations begin to appear in the s-e diagram, indicating the formation of a macro-crack or fiber breakage. After exceeding the maximum bending force F_tb_, the strain-control range (ε_tb_-ε_d_) begins—called the rope effect. In this range, strains increase, macro-cracks open, additional fibers break (Figure 7), and the bending force begins to decrease. The end of this phase is assumed to be the deformation value for which the force value is equal to the force value that determined the formation of the first crack—ε_d_ in Figure 7. This phase is followed by the crack propagation phase (ε_d_-ε_px_). The value of ε_px_ is determined for a force corresponding to ½ the force value at point ε_d_.

**Figure 8:**
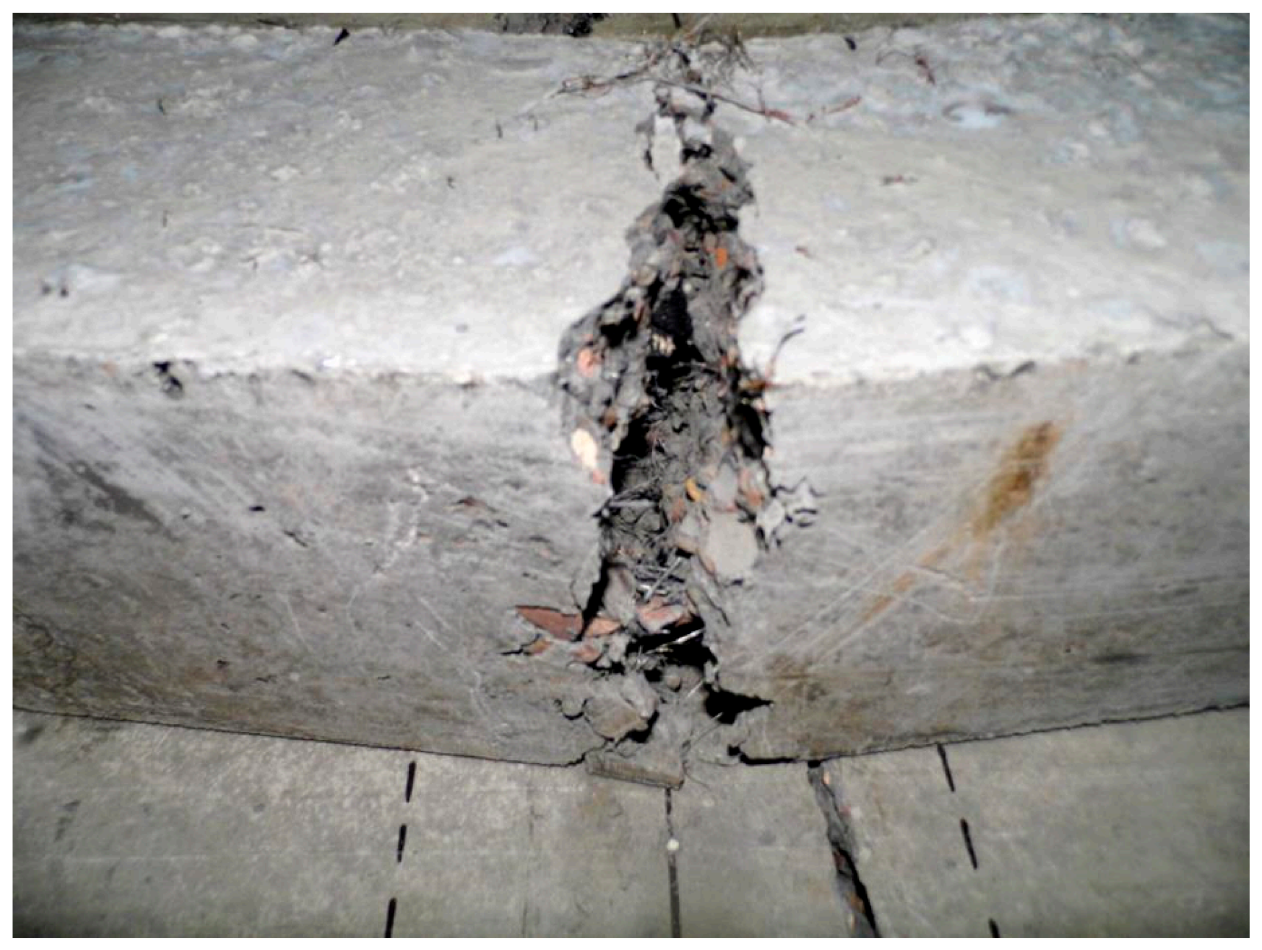
Progressive crack opening during a 4-point bending test of a 150 × 150 × 600 mm beam (span between the support axes L = 450 mm).

Progressive crack opening and fiber breakage can be observed in Figure 8. Results of flexural tensile strength tests (four-point bending, 150 × 150 × 600 mm beams) performed in individual series are juxtaposed in Table 7.

Explanation of the labels in Figure 7

f_0_—failure of the concrete matrix (without fibers);
F_0_—destructive force on the concrete specimen (matrix);
ε_0_—strain of the concrete specimen (matrix) at the moment of failure;
W_0_—work of destruction of the matrix;
f_cr_—initiation of the first crack in the composite specimen;
F_cr_—force causing the first crack in the tested specimen;
W_cr_—work causing cracking;
ε_cr_—strain at the moment of cracking;
f_tb_—maximum strength of the specimen;
F_tb_—maximum destructive force;
ε_s_—strain in the strengthening phase;
W_S_—work in the strengthening phase;
f_d_—end of the strain-control phase;
F_d_—force equal to F_cr_—defining the end of the strain-control phase;
ε_d_—strain in the strain-control phase;
W_D_—work in the strain-control phase;
f_px_—end of the crack propagation phase; point determined for F_px_ = ½F_d;_
ε_p_—strain in the crack propagation phase;
W_p_—work in the crack propagation phase;
A_0_—work range of the concrete matrix until failure;
A_F_—elastic work range (within the scope of Hooke’s law);
A_S_—strengthening range;
A_D_—strain-control range;
A_p_—crack propagation range;
f_c_—compressive strength;
f_t_—flexural tensile strength;
$k_{0}=\frac{f_{t}}{f_{c}}$—coefficient defining the brittleness of the concrete matrix;
$k=\frac{f_{t,fib}}{f_{c}}$—coefficient defining the brittleness of fiber-reinforced concrete;
$k^{0}=\frac{k_{0}}{k}$—ratio of brittleness of the matrix to that of fiber-reinforced concrete.

Each characteristic point of the stress–strain curve is a function of three values: deflection, load, and work: *f*_x_ = *f*_x_ (*F*_x_; *ε*_x_; *W*_x_).

concrete matrix: *A*_0_ (*ε*_0_; F_0_; $W_{0}=\int_{0}^{\epsilon_{0}}\;Fd\varepsilon$) ⇔ f_0_ (*ε*_0_; *F*_0_; *W*_0_);

first crack: *A*_F_ (*ε*_cr_; F_cr_; $W_{cr}=\int_{0}^{\epsilon_{cr}}\;Fd\varepsilon$) ⇔ *f*_cr_ (*ε*_cr_; *F*_cr_; *W*_cr_);

strengthening: *A*_S_ (*ε*_tb_; *F*_tb_; $W_{S}=\int_{\epsilon_{cr}}^{\epsilon_{tb}}\;Fd\varepsilon =W_{tb}-W_{cr}$) ⇔ *f*_tb_ (*ε*_tb_; *F*_tb_; *W*_tb_);

deflection control

*A*_D_ (*ε*_td_; *F*_d_; $W_{D}=\int_{\epsilon_{tb}}^{\epsilon_{b}}\;Fd\varepsilon =W_{d}-W_{tb}$) ⇔ *f*_d_ (*ε*_d_; *F*_d_; *W*_d_);

crack propagation:

*A*_p_ (*ε*_px_; *F*_px_; $W_{D}=\int_{\epsilon_{b}}^{\epsilon_{px}}\;Fd\varepsilon =W_{px}-W_{d}$) ⇔ *f*_px_ (*ε*_px_; *F*_px_; *W*_px_);

*f*_x/0_—change of the value in point *f*_x_ relative do the matrix point *f*_0_:

*f*_x/0_ (*ε*_x_/*ε*_0_; *F*_x_/*F*_0_; *W*_x_/*W*_0_);

*A*_X/0_—Change of the area under the curve (work) *A*_X_ between points *f*_x1_ and *f*_x2_, relative to the area under the matrix curve *A*_0_:

*A*_x/0_ [(*ε*_x2_ − *ε*_x1_)/*ε*_0_; *F*_x2_/*F*_0_; (*W*_x2_ − *W*_x1_)/*W*_0_].

Comparisons should be made of the strengthening effects *f*_x_, *A*_X_ relative to the concrete matrix (*f*_x_/0, *A*_X/0_), and relative to the first-crack values (*f*_x/cr_; *A*_X/cr_) at the characteristic points of the curve according to the following relation:

- First crack:

f^o^_cr_ (ε_cr_/ε_0_; F_cr_/F_0_; W_cr_/W_0_) ⇔ A^o^_F_ (ε_cr_/ε_0_; F_cr_/F_0_; W_cr_/W_0_);

- Strengthening:

f^cr^_tb_ (ε_tb_/ε_cr_; F_tb_/F_cr_; W_tb_/W_cr_) ⇔ A^o^_S_ ((ε_tb_ − ε_cr_)/ε_0_; F_tb_/F_0_; (W_tb_ − W_cr_)/W_0_);

- Deflection control:

f^cr^_d_ (ε_d_/ε_cr_; F_d_/F_cr_; W_d_/W_cr_) ⇔ A^o^_D_ ((ε_d_ − ε_tb_)/ε_0_; F_d_/F_0_; (W_d_ − W_tb_)/W_0_);

- Crack propagation:

f^cr^_px_ (ε_px_/ε_cr_; F_px_/F_cr_; W_px_/W_cr_) ⇔ A^o^_px_ ((ε_px_ − ε_D_)/ε_0_; F_px_/F_0_; (W_px_ − W_D_)/W_0_).

**Figure 9:**
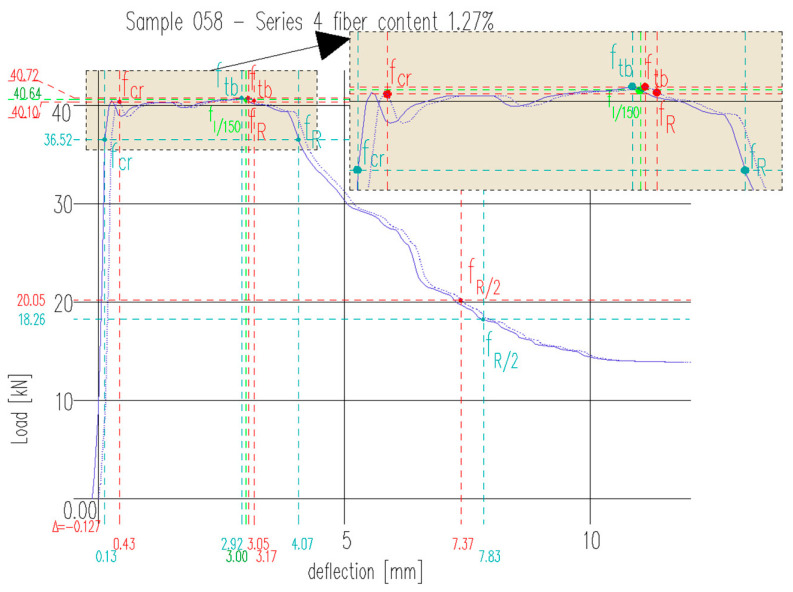
Example of a deformation graph for one of the tests with characteristic points marked.

The graph (Figure 9) demonstrates the qualitative adhesion of the fibers to the concrete matrix. The force drops visible on the graph with progressive deformation indicate either the opening of new cracks or fiber breakage. A graph without significant force drops (close to linear) indicates fiber pullout caused by their low adhesion to the concrete matrix.

Values of the characteristic points of the FSD/ESD method (cf. Figure 8) determined from the corrected load–deflection curves for beams exhibiting strengthening: deflection Δ [mm], force F [kN] and work W [J]; k—brittleness coefficient for the matrix rows; the value corresponds to k_0_. All data are given in Appendix A for clarity of presentation.

## 4. Analysis of Results and Brief Discussion

**Figure 10:**
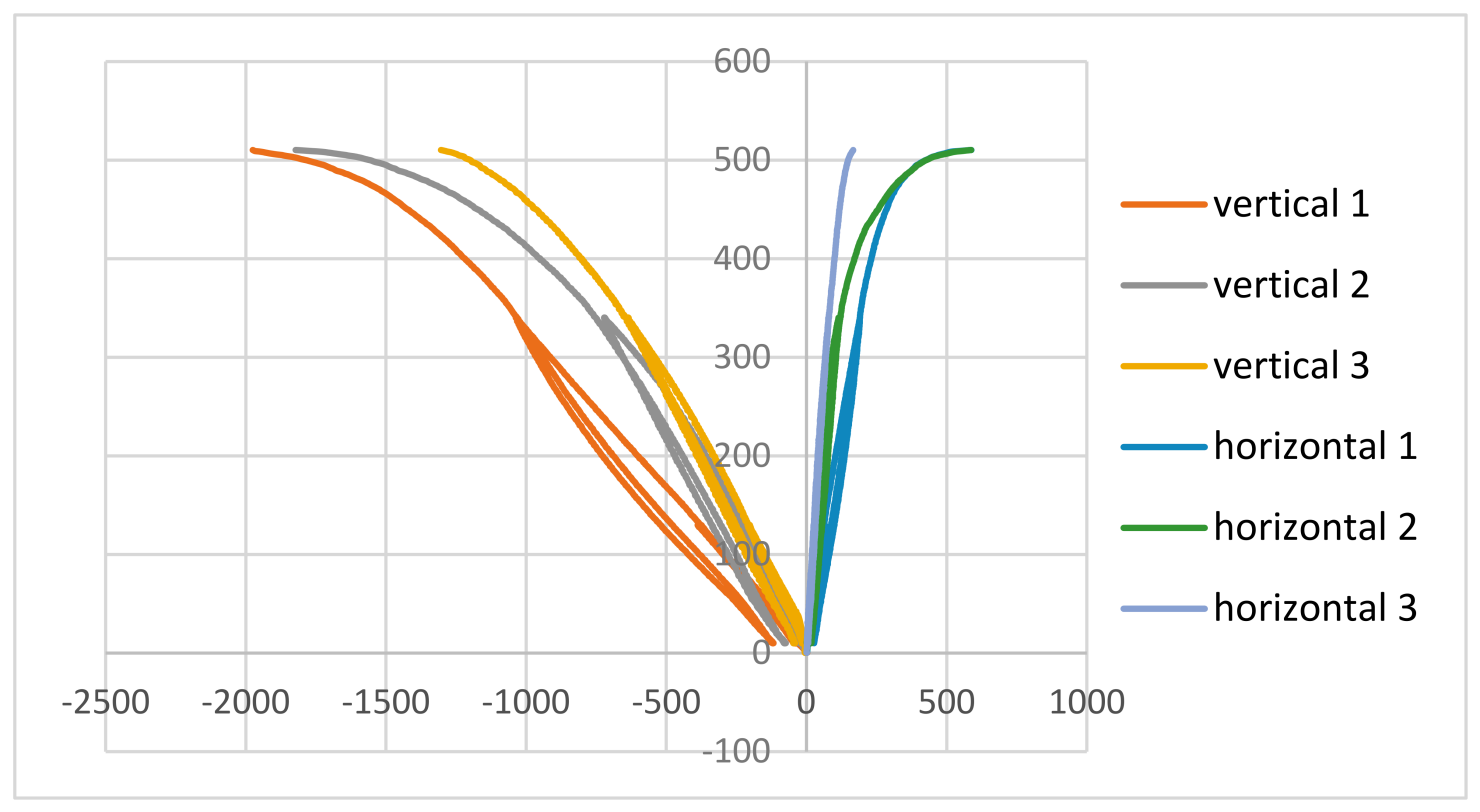
Examples of sample deformations measured on individual strain gauges. Readings with a negative sign indicate Young’s modulus, and readings with a positive sign indicate Poisson’s ratio.

The results presented above clearly demonstrate the significant effect of adding the fibrous phase with rubber impurities on the magnitude of deformation transverse to the direction of force application. This appears to be caused by the addition of rubber impurities to the matrix, which are characterized by high compressibility compared to the aggregate, which, given the sample deformation in the direction transverse to the force application is approximately seven times smaller than the deformation in the direction of force application, appears to be of significant importance. Figure 10 presents graphs of the measured sample deformations on individual strain gauges. The acting force [kN] is plotted on the vertical axes, and the sample deformation [μm/m] is plotted on the horizontal axes.

Measuring sample deformation during testing is difficult due to manufacturing constraints and technical and measurement limitations. The beam deflection tests presented in this study are also subject to error. The beams were tested by subjecting them to four-point bending without a notch. The lack of a notch itself caused random crack formation (in various locations)—not always at the center of the beam’s span, the location where the deformations were measured. Deflections under the crack, which occurred at a location other than the center of the beam’s span, were relatively greater than those measured at the center of the span.

Many standards recommend testing deflection relative to the beam’s neutral axis. However, this testing method requires a notch in the beam to force a crack to form in a predetermined location. The idea behind FSD/ESD is to more optimally utilize the cross-section of a quasi-brittle composite, so notching the sample contradicts its purpose. A recurring error made by the authors (in all tested samples), due to technical constraints and assumed to be relatively small, is the failure to account for the settlement of the tested samples at their supports. Almost every analyzed test method appears to introduce measurement errors, varying depending on the method chosen. The ideal solution, suggested by Logoń, would be to perform multi-point measurements using two cameras linked to a press and a computer equipped with software capable of recording sample deformation at several points along the beam’s span and above each of its supports. Placing cameras on both sides of the tested sample would allow for the detection of any torsion during testing. Based on these determined deformations, it is possible to actually determine the beam’s deflection along its length (including under the crack), without forcing the crack to form—thus disrupting the actual performance of the tested element. All samples tested in this study are subject to the same errors resulting from imperfect strain measurements. Therefore, it was concluded that their influence can be ignored at this stage of the discussion, assuming that its value is similar for all tested samples.

**Figure 11:**
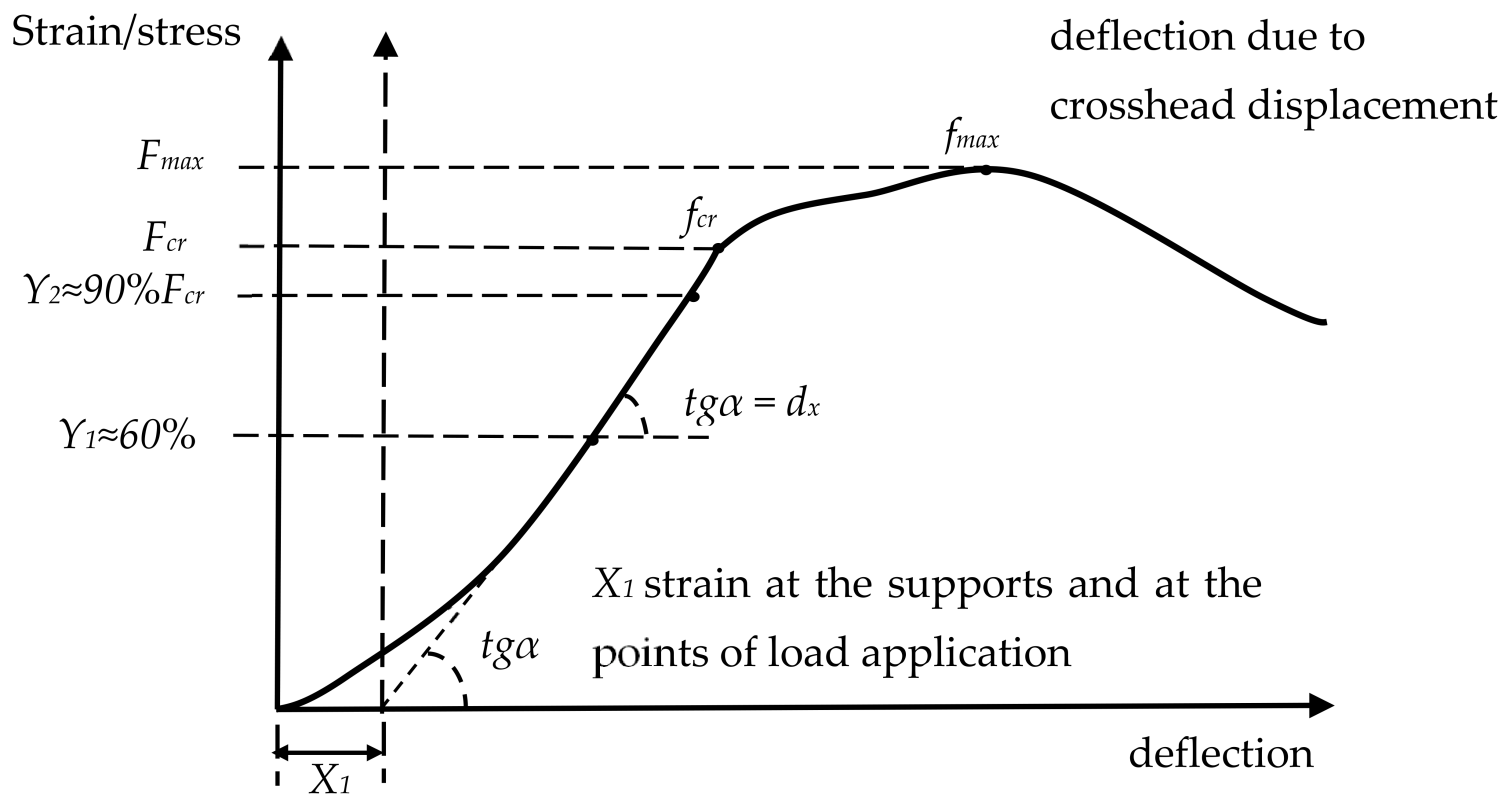
Load-deformation graph, determining deflection and deformation capacity from the crosshead [60].

Another approximation is the assumption of linearity of the graph within the range of Hooke’s law. This lack of linearity results in the aforementioned errors during sample strain measurements. Analyzing numerous scientific publications, it was noted that in most of them, this nonlinearity was neglected. This may be due to the lack of a standardized method for correcting the graphs. It should be remembered that failure to correct the obtained graphs also affects the results of strength calculations of composite materials, which start from the sample deflection and read the corresponding previously described areas under the graphs (e.g., ASTM C1018 [59]). Logoń suggested a solution to this problem in his presentation from 2016 [60]. He noticed a certain dependence in the nonlinearity of the graph within the scope of Hooke’s law. According to Logoń’s observations, the σ-ε curve takes a rectilinear form in the range Y_1r_–Y_2_ (Figure 11), where Y_1_–Y_2_ ≈ 60% F_cr_–90% F_cr_ (dependent on the composite reinforcement method, sample loading rate, test type, etc.). He suggested replacing the nonlinear part of the 0–Y_1_ graph with a rectilinear segment resulting from the extension of the Y_1_–Y_2_ segment to the x-axis, and the intersection point marks the beginning of the measurement. The determined tan α is the modulus of elasticity of the tested composites. An explanation of the idea is presented in Figure 11.

Furthermore, analyzing the obtained graphs revealed another important aspect for the study and the accuracy of the results. Despite great care in observing the first crack and the force causing it, it was noted that the reading of the cracking force F_cr_ in many cases was already in the nonlinear sections of the graph, characteristic of exceeding the range of Hooke’s law. This indicates that micro-cracks and accompanying permanent deformations occurred within the tested samples before the macro-crack appeared on the sample surface. Therefore, in the author’s opinion, the requirement to read the F_cr_ force directly from the graph or using another diagnostic method (e.g., acoustic methods) should be added to the above, and not, as suggested in numerous publications, based on observation of the sample surface. Observation can be helpful in evaluating the test results, but it should not be accepted uncritically during the development of test results.

It should be noted that each series was made of a separate batch of concrete prepared on a different date: series 1, 1a and 2 were made of ready-mixed concrete (series 1 and 1a—grade C20/25, series 2—grade C45/55), whereas series 3 and 4 were laboratory mixes based on slowly hardening CEM III/A 32.5 cement (Section 2.3). For this reason, the absolute strength of the plain matrix (0.00% of fibers) differs between the individual series and testing ages, and the effect of the fiber addition should be evaluated by comparison with the reference samples (0.00% of fibers) of the same series and age. In the strong matrix of series 2, the fiber addition slightly reduced the average compressive strength (at 28 days from 69.5 MPa for the plain matrix to 61.5 MPa for 0.40% of fibers), which is a well-known effect attributed mainly to the more difficult compaction of the fresh mix containing irregular recycled fibers. The decrease in compressive strength observed in some series after the addition of fibers (e.g., series II: 69.5→61.5 MPa) is probably a well-known compromise in the literature—uncleaned fibers worsen the density of the mixture.

In the weak, slowly hardening matrix of series 3, on the other hand, the fiber addition clearly increased the compressive strength (from 12.8 to 19.7 MPa at the age of 35 days). The moderate decrease observed for strong matrices is compensated by the substantial improvement in the post-cracking flexural behavior described in Section 3.3 and Section 3.4.

## 5. Conclusions

The main utility of this work is to demonstrate the practical possibilities of recycling used tires, particularly for the production of fiber-reinforced concrete. Based on the results of numerous test series, the feasibility of using the described wire for reinforcing concrete for specific applications was demonstrated. The conducted tests allowed for the formulation of conclusions with practical applications in concrete construction.

- Steel fibers from recycled car tires (RFRC—Recycled Fiber Reinforced Concrete) are an effective method for strengthening the strength of concrete elements under uniaxial stress and increasing their resistance to brittle fracture. The observed increase in brittle fracture strength was nearly 400% of the matrix strength.
- Steel reinforcement contaminated with rubber from tire recycling, used to reinforce concrete, improves the strength parameters of tensile concrete and significantly increases the range of plastic deformation after failure of the composite element, which impacts the safety of the structure. The addition of recycled fibers leads to a uniform compressive strength of the fiber-enriched concrete compared to its concrete matrix (the coefficients of variation for the fiber-enriched concrete are lower than for the unenriched samples). The failure mechanism of the fiber-enriched samples, unlike the concrete samples, is not explosive.

Samples with a high fiber addition (approximately 1.3%) exhibited a higher flexural tensile strength than the concrete samples—a nearly two-fold increase in strength was observed. Even more pronounced benefits are observed in the case of weaker concrete (grades lower than C20/25). The failure mechanism of the fiber-enriched samples is an undoubted advantage. After the fiber-enriched concrete fails, the load transferred by the element decreases with increasing plastic strain (rope effect).

Small additions of recycled fibers (less than 0.15%) resulted in a decrease in flexural tensile strength of approximately 30%. The addition of steel fibers (contaminated with rubber) to approximately 1.3% reduces the shrinkage of concrete with these fibers by 7–10% compared to a fiber-free concrete matrix cured under the same conditions.

The observed relationship may be applicable to concrete intended to have increased resistance to potential acts of vandalism, for example, for enclosures with increased burglary resistance.

Furthermore, certain relationships were observed that, in the authors’ opinion, require more research. These studies should include:

- Studies of Poisson’s ratio of concrete and fiber-reinforced concrete, considering the variation in recycled fiber content and the strength of the concrete matrix.
- Studies of the creep of concrete and fiber-reinforced concrete in the direction perpendicular to the force applied.
- Extensive studies of the shrinkage of concrete and fiber-reinforced concrete. These studies should consider various shapes and operating conditions of the components (temperature, air humidity, surfaces from which water can evaporate).
- Investigating the effect of adding recycled wire reinforcement on punching shear strength.
- Investigating how concrete formulation affects the load-bearing capacity of the reinforcement.

In the authors’ opinion, the right direction would be to prepare and implement an experimental research program conducted on small- and large-sized concrete elements reinforced with recycled fibers, allowing for the formulation of an algorithm for analytically estimating the parameters of hardened concrete reinforced with recycled fibers, considering rheological processes.

The tests were performed on 281 samples in five series. The testing standards were based on the recommendations of Eurocode 2. Deliberately simple concrete mix formulations were adopted, as the technology of this type of fiber concrete assumes its use in concrete structures of lower standards. Selected test results are presented for different concrete strengths in order to demonstrate the sensitivity of the strength parameters to the random character of the applied fibers. An extensive report on these tests and detailed conclusions is contained in [56].

## Figures and Tables

**Table 1:** Results of counting individual wires in the cut planes of the samples (series 1 and 2).

	Amount of Fibers [%]	Amount in Cross-Section 1 [items]	Amount in Cross-Section 2 [Items]	Results for a Single Sample			Results for Group of Samples		
				Average [Items]	Standard Deviation [Items]	Coefficient of Variation [%]	Average [Items]	Standard Deviation [Items]	Coefficient of Variation [%]
1	0.33	205	267	236	31	13.14	186.83	74.35	39.79
		191	162	176	14	8.22			
		166	130	148	18	12.16			
2	0.13	107	131	118	12	9.70	170.01	103.68	60.98
		142	164	153	11	7.19			
		185	292	238	53	22.43			
	0,27	202	172	187	15	8.02	203.83	37.90	18.59
		219	186	202	16	8.15			
		240	204	222	18	8.11			
	0.40	193	210	201	8	4.22	221.02	55.24	25.01
		240	228	234	6	2.56			
		179	276	227	48	21.32			

**Table 2:** Average values of the tensile strength of the wires and their elongation at break.

	Diameter [mm]	F_max_ [N]	ΔL (F_max_) [mm]	F_ult_ [N]	ΔL (F_ult_) [mm]	R_m_ [MPa]	A [%]
2	0.29	145	0.70	-	-	2195.24	1.8
3	0.30	158	0.70	-	-	2235.24	1.8
4	0.90	1120	2.70	982	2.70	1760.53	6.8
5	0.90	1250	1.80	879	2.10	1964.88	4.5
6	1.10	1350	1.60	-	-	1420.56	4.0

Two types of bars were distinguished: 0.3 and 0.9 diameters. A—elongation at break, F_max_—maximum recorded force, F_ult_—ultimate force, R_m_—steel strength.

**Table 3:** Results of concrete compressive tests performed in individual series 1 and 1a.

Series	Time	Amount of Fibers	Symbol	Strength	Average Strength	Standard Deviation	Coefficient of Variation
	[days]	[%]		[MPa]	[MPa]	[MPa]	%
1	42	0.00	B01	32.2			
			B02	30.4	30.6	1.46	4.78
			B03	29.3			
		0.33	D01	25.8			
			D02	26.0	26.0	0.20	0.77
			D03	26.2			
1a	4	0.00	B1	9.3			
			B2	9.3	9.2	0.23	2.52
			B3	8.9			
		0.33	D1	10.2			
			D2	9.8	10.2	0.45	4.41
			D3	10.7			
	7	0.00	B4	14.7			
			B5	15.1	14.7	0.59	4.00
			B6	14.9			
		0.33	D4	14.7			
			D5	13.8	14.5	0.67	4.58
			D6	15.1			
	14	0.00	B7	20.4			
			B8	19.6	19.9	0.42	2.09
			B9	19.8			
		0.33	D7	21.3			
			D8	20.2	20.8	0.56	2.68
			D9	20.9			
	28	0.00	B10	29.6			
			B11	29.8	30.2	0.81	2.70
			B12	31.1			
		0.33	D10	30.9			
			D11	29.6	30.5	0.75	2.46
			D12	30.9			

**Table 4:** Results of concrete compressive tests performed in individual series 2 and 3. Each series is a separate batch of concrete.

Series	Time	Fibers	Symbol	Volume	Density	Average Density	Strength	Average Strength	Standard Deviation	Coefficient of Variation
	[Days]	[%]		[cm^3^]	[kg/m^3^]	[kg/m^3^]	[MPa]	[MPa]	[MPa]	%
2	3	0.00	001	3364	2450.7	2446.4	47.4	42.30	7.86	18.58
			002	3345	2452.1		46.3			
			003	3356	2436.4		33.2			
		0.13	016	3391	2382.3	2400.4	43.6	41.87	1.66	3.97
			017	3398	2403.2		40.3			
			018	3365	2415.7		41.7			
		0.27	028	3390	2440.6	2434.5	41.7	40.53	3.87	9.56
			029	3375	2440.1		43.7			
			030	3368	2422.8		36.2			
		0.40	045	3401	2411.6	2412.6	39.8	39.30	2.00	5.10
			046	3414	2416.4		41.0			
			048	3414	2409.9		37.1			
	7	0.00	004	3346	2446	2448.9	60.4	58.3	1.87	3.21
			005	3374	2457.1		57.0			
			006	3375	2443.6		57.3			
		0.13	019	3382	2417.3	2431.9	54.7	51.5	3.64	7.06
			020	3377	2415.8		52.3			
			021	3361	2462.6		47.6			
		0.27	027	3386	2435.6	2438.2	48.7	47.0	2.55	5.42
			031	3396	2442.1		48.2			
			032	3391	2437		44.1			
		0.40	042	3376	2411.5	2420.2	46.3	49.1	2.52	5.13
			043	3370	2422		51.2			
			044	3407	2427		49.8			
	28	0.00	010	3365	2432.3	2445.4	73.7	69.5	4.86	6.99
			011	3349	2459.4		70.6			
			012	3353	2444.5		64.2			
		0.13	022	3371	2435.9	2434.6	67.5	64.4	2.87	4.47
			023	3329	2451.4		61.8			
			024	3369	2416.6		63.8			
		0.27	033	3398	2445	2442.7	67.1	66.6	3.78	5.67
			034	3372	2442.8		70.1			
			035	3409	2440.3		62.6			
		0.40	040	3379	2425.2	2423.2	61.2	61.5	2.54	4.13
			041	3362	2420.8		64.2			
			047	3368	2423.6		59.1			
3	35	0.00	B1	3301	2264.2	2255.6	12.4	12.8	0.81	6.37
			B2	3328	2259.8		13.1			
			B3	3265	2273.6		13.4			
			B4	3265	2234.2		11.5			
			B5	3329	2246.3		12.0			
		1.27	D1	3290	2242.3	2251.1	19.4	19.7	0.73	3.72
			D2	3357	2252.6		20.0			
			D3	3355	2250.5		19.5			
			D5	3330	2244.6		19.1			
			D6	3305	2265.4		20.4			

**Table 5:** Results of concrete compressive tests performed in individual series 4.

Series	Time	Fibers	Symbol	Volume	Density	Average Density	Strength	Average Strength	Standard Deviation	Coefficient of Variation
	[Days]	[%]		[cm^3^]	[kg/m^3^]	[kg/m^3^]	[MPa]	[MPa]	[MPa]	%
4	35	0.00	001	3355	2315.6		34.8	33.7	1.07	3.17
			002	3354	2301.4	2304.1	33.5			
			003	3329	2295.3		32.7			
		0.25	007	3354	2318.1		32.0	31.9	0.88	2.74
			008	3364	2333.1	2320.9	32.8			
			009	3352	2311.6		31.0			
		0.50	013	3364	2320.1		30.3	28.9	2.64	9.11
			016	3394	2230.0	2291.9	25.9			
			017	3356	2325.6		30.6			
		0.76	020	3370	2315.7		31.0	31.3	0.57	1.83
			023	3417	2336.2	2327.8	30.9			
			024	3285	2331.3		31.9			
		1.27	033	3409	2315.4		34.3	33.3	0.94	2.82
			034	3390	2312.5	2318.1	33.0			
			036	3380	2326.4		32.5			

**Table 6:** Results of brittle fracture resistance tests of concrete.

Series	Fibers [%]	Series I Specimens: Cylinders ø113, l = 300. Series III and IV Specimens: Cubes 150 × 150 × 150							
		Symbol	Volume cm^3^	Density kg/m^3^	Average Density [kg/m^3^]	Strength f_ci_ [MPa]	Average Strength f_cm_ [MPa]	Standard Deviation s_fc_	Coefficient of Variation v_fc_ [%]
I	0	WB01	3518	2449	2453	2.9	3.0	0.3	7.5
		WB02	3527	2463		3.3			
		WB03	3530	2447		2.8			
	0.4	WD01	3517	2458	2479	3.5	3.6	0.1	2.6
		WD02	3528	2485		3.5			
		WD03	3502	2493		3.7			
III	0	B6	3303	2215	2240	0.48	0.522	0.1	23.7
		B7	3277	2259		0.65			
		B8	3306	2246		0.43			
		B9	3354	2216		0.57			
		B10	3330	2263		0.48			
	1.27	D4	3309	2236	2248	2.07	2.018	0.2	10.9
		D7	3346	2266		2.18			
		D8	3368	2247		2.06			
		D9	3346	2252		2.02			
		D10	3316	2239		1.76			
IV	0	004	3359	2306	2304	2.1	1.7	0.3	19.2
		005	3336	2305		1.6			
		006	3272	2301		1.5			
	0.25	010	3357	2326	2317	2.2	2.1	0.1	5.2
		011	3362	2318		2.2			
		012	3359	2306		2.0			
	0.5	014	3396	2336	2326	2.4	2.0	0.3	16.8
		015	3341	2318		1.8			
		018	3341	2324		1.9			
	0.76	019	3378	2327	2332	2.4	2.3	0.3	11.2
		021	3362	2346		2.6			
		022	3381	2322		2.0			
	1	026	3269	2333	2317	3.4	3.5	0.1	2.7
		027	3312	2325		3.5			
		028	3302	2291		3.6			
	1.25	031	3375	2326	2322	3.1	3.0	0.3	10.3
		032	3355	2316		2.7			
		035	3388	2325		3.3			

**Table 7:** Results of flexural tensile strength tests (four-point bending, 150 × 150 × 600 mm beams).

Series	Fibers [%]	Symbol	Density [kg/m^3^]	Average Density [kg/m^3^]	F_cr_ (Observed) [kN]	Fult [kN]	f_t,flex_ [MPa]	Average f_t,flex_ [MPa]	Standard Deviation [MPa]	Coefficient of Variation [%]
I	0.00	I	2236.6	2211.0	–	–	–	4.4	0.2	4.1
		II	2195.4		–	33.54	4.3			
		III	2201.1		–	35.25	4.6			
	0.33	IV	2224.2	2211.6	–	28.58	3.7	3.8	0.4	10.7
		V	2214.3		–	33.67	4.3			
		VI	2196.3		–	27.42	3.5			
II	0.00	064	2395.5	2391.6	–	74.89	9.8	9.1	0.7	8.0
		067	2391.4		–	69.78	9.0			
		068	2387.9		-	64.97	8.4			
	0.13	070	2359.6	2359.0	39.05	40.09	5.3	5.6	4.1	72.4
		072	2351.1		74.24	75.94	9.9			
		073	2366.4		76.37	77.30	1.7			
	0.27	075	2364.6	2381.7	65.33	68.76	9.0	9.6	0.5	5.7
		076	2402.5		76.29	76.51	9.9			
		077	2378.0		77.30	77.81	10.0			
	0.40	079	2365.9	2373.6	65.90	67.20	8.5	8.3	1.0	12.4
		080	2376.0		69.93	67.81	9.2			
		083	237.9		51.79	55.63	7.1			
III	0.00	BB1	2264.7	2265.9	–	17.87	2.3	2.4	0.4	17.4
		BB2	2274.4		–	18.78	2.4			
		BB3	2266.7		–	19.19	2.5			
		BB4	2249.8		–	20.98	2.8			
		BB5	2277.2		–	15.43	2.1			
		BB6	2262.5		–	16.12	2.1			
	1.27	BD1	2303.7	2313.8	28.11	32.37	4.2	4.6	2.1	45.1
		BD2	2345.3		29.21	48.08	7.1			
		BD3	2297.6		26.90	31.88	4.0			
		BD4	2316.0		27.98	29.57	3.8			
		BD5	2309.1		29.26	37.56	4.9			
		BD6	2311.1		20.37	27.24	3.6			
IV	0.00	037	2325.7	2333.8	–	30.65	4.0	4.5	0.4	8.9
		038	2334.2		–	35.15	4.6			
		039	2338.7		–	37.36	4.8			
	0.25	041	2301.1	2305.0	23.40	26.32	3.4	3.6	0.4	11.0
		042	2297.8		30.40	31.38	4.1			
		044	2316.1		22.80	25.71	3.3			
	0.50	045	2312.3	2311.0	23.70	27.94	3.6	3.8	0.2	4.8
		046	2312.7		27.20	29.10	3.7			
		047	2308.1		27.70	30.42	4.0			
	0.76	049	2332.6	2325.6	35.00	36.24	4.6	4.2	0.4	8.6
		050	2318.8		29.00	32.58	4.1			
		051	2325.4		28.00	29.48	3.9			
	1.00	053	2321.9	2292.4	34.80	37.33	4.8	4.3	0.4	8.5
		054	2278.8		32.00	33.36	4.2			
		055	2276.5		30.40	32.94	4.0			
	1.27	057	2329.7	2329.1	36.20	37.72	4.8	4.7	0.4	8.7
		058	2309.0		40.1	40.72	5.1			
		059	2348.5		33.00	34.88	4.2			

## Data Availability

The original contributions presented in this study are included in the article. Further inquiries can be directed to the corresponding author.

## Appendix A

The table below shows clearly that making readings without preliminary preparation of graphs, and taking the drawing force value on the basis of observations, leads to the formation of large errors. Therefore, it is considered necessary to correct the graphs obtained from the testing machines, and the observation of the drawing force values should only be a check of the correctness of the readings made directly from the graphs.

**Table A1:** Values of the characteristic points of the FSD/ESD method determined from the corrected load–deflection curves for beams exhibiting strengthening: deflection Δ [mm], force F [kN], and work W [J]; k—brittleness coefficient for the matrix rows; the value corresponds to k_0_.

Series	Specimen Designation	Characteristic Points																									
		Diagrams Corrected According to Figure 11 (Cracking Force Read from the Diagrams)								Diagrams Obtained from Testing Machines (Cracking Force Based on Observation During the Test								Difference in Readings Between Corrected and Uncorrected Diagrams								Brittleness	
		Char. pt.	[mm]			[kN]	[J]				[mm]			[kN]	[J]				[mm]			[kN]	[J]			k	k0
II	concr. average	A_0_ = f_0_	(	0.3	;	69.9	;	10.4	)	A_0_ = f_0_	(	0.3	;	69.9	;	10.4	)	A_0_ = f_0_	(	0.0	;	0.0	;	0.0	)	0.2	x
	075	A_0cr_	(	0.0	;	13.5	;	2.4	)	A_0cr_	(	1.1	;	1.0	;	1.1	)	A_0cr_	(	−1.1	;	12.5	;	1.3	)	0.2	1.0
		A_0M_	(	x	;	x	;	x	)	A_0M_	(	0.1	;	1.0	;	0.1	)	A_0M_	(	x	;	x	;	x	)		
		A_0Rp_	(	x	;	x	;	x	)	A_0Rp_	(	2.2	;	0.9	;	2.2	)	A_0Rp_	(	x	;	x	;	x	)		
		A_0Pr_	(	0.0	;	6.8	;	13.3	)	A_0Pr_	(	0.6	;	0.5	;	0.9	)	A_0Pr_	(	−0.6	;	6.3	;	12.4	)		
		∑		0.0		∑		15.7		∑		∑		∑		4.3		∑		−1.7		∑		13.7			
		f_cr_	(	0.3	;	68.8	;	12.2	)	f_cr_	(	0.3	;	65.3	;	11.3	)	f_cr_	(	0.0	;	2.4	;	0.9	)		
		f_tb_	(	x	;	x	;	x	)	f_tb_	(	0.4	;	68.8	;	12.2	)	f_tb_	(	x	;	x	;	x	)		
		f_R_	(	x	;	x	;	x	)	f_R_	(	1.1	;	65.3	;	35.1	)	f_R_	(	x	;	x	;	x	)		
		f_R_/2	(	1.2	;	34.4	;	12.2	)	f_R_/2	(	1.3	;	33.2	;	44.8	)	f_R_/2	(	−0.1	;	1.2	;	−32.5	)		
	076	A_0cr_	(	0.0	;	15.0	;	0.9	)	A_0cr_	(	1.1	;	1.1	;	0.7	)	A_0cr_	(	−1.1	;	13.9	;	0.1	)	0.2	1.1
		A_0M_	(	0.0	;	15.1	;	5.2	)	A_0M_	(	1.2	;	1.1	;	2.8	)	A_0M_	(	−1.2	;	14.0	;	2.4	)		
		A_0Rp_	(	0.0	;	15.0	;	8.1	)	A_0Rp_	(	0.8	;	1.1	;	2.0	)	A_0Rp_	(	−0.8	;	13.9	;	6.1	)		
		A_0Pr_	(	0.0	;	7.5	;	13.2	)	A_0Pr_	(	0.9	;	0.6	;	1.4	)	A_0Pr_	(	−0.9	;	7.0	;	11.8	)		
		∑		0.0		∑		27.3		∑		4.0	∑	∑		6.9		∑		−4.0		∑		∑			
		f_cr_	(	0.1	;	76.3	;	4.5	)	f_cr_	(	0.3	;	76.3	;	7.7	)	f_cr_	(	−0.2	;	0.0	;	−3.2	)		
		f_tb_	(	0.4	;	76.5	;	26.3	)	f_tb_	(	0.7	;	76.5	;	36.6	)	f_tb_	(	−0.3	;	0.0	;	−10.3	)		
		f_R_	(	0.6	;	76.3	;	41.1	)	f_R_	(	1.0	;	76.3	;	57.1	)	f_R_	(	−0.4	;	0.0	;	−16.0	)		
		f_R_/2	(	1.0	;	38.2	;	65.9	)	f_R_/2	(	1.3	;	38.1	;	71.4	)	f_R_/2	(	−0.3	;	0.0	;	−4.5	)		
	077	A_0cr_	(	0.0	;	13.8	;	2.7	)	A_0cr_	(	0.6	;	1.1	;	0.6	)	A_0cr_	(	−0.6	;	12.7	;	2.1	)	0.2	1.1
		A_0M_	(	0.0	;	15.3	;	7.2	)	A_0M_	(	0.1	;	1.1	;	0.1	)	A_0M_	(	−0.1	;	14.2	;	7.0	)		
		A_0Rp_	(	0.0	;	13.8	;	20.2	)	A_0Rp_	(	3.8	;	1.0	;	9.1	)	A_0Rp_	(	−3.8	;	12.8	;	11.0	)		
		A_0Pr_	(	0.0	;	6.9	;	22.6	)	A_0Pr_	(	0.7	;	0.5	;	1.2	)	A_0Pr_	(	−0.7	;	6.4	;	21.4	)		
		∑		0.1		∑		52.6		∑		5.2	∑	∑		11.0		∑		−5.1		∑		∑			
		f_cr_	(	0.4	;	70.3	;	13.7	)	f_cr_	(	0.2	;	70.3	;	6.0	)	f_cr_	(	0.2	;	0.0	;	7.7	)		
		f_tb_	(	0.8	;	77.8	;	36.5	)	f_tb_	(	0.2	;	77.8	;	7.4	)	f_tb_	(	0.6	;	0.0	;	29.1	)		
		f_R_	(	1.4	;	70.3	;	102.5	)	f_R_	(	1.4	;	70.3	;	102.3	)	f_R_	(	0.0	;	0.0	;	0.2	)		
		f_R_/2	(	1.6	;	35.2	;	114.7	)	f_R_/2	(	1.6	;	35.2	;	114.6	)	f_R_/2	(	0.0	;	0.0	;	0.2	)		
III	concr. average	A_0_ = f_0_	(	0.3	;	18.1	;	5.2	)	A_0_ = f_0_	(	0.3	;	18.1	;	5.2	)	A_0_ = f_0_	(	0.0	;	0.0	;	0.0	)	0.1	x
	BD1	A_0cr_	(	0.0	;	13.8	;	1.4	)	A_0cr_	(	1.9	;	1.8	;	1.9	)	A_0cr_	(	−1.9	;	12.0	;	−0.6	)	0.2	2.1
		A_0M_	(	0.1	;	17.0	;	10.2	)	A_0M_	(	2.6	;	1.8	;	4.9	)	A_0M_	(	−2.5	;	15.2	;	5.3	)		
		A_0Rp_	(	0.2	;	13.8	;	39.6	)	A_0Rp_	(	3.8	;	1.6	;	7.0	)	A_0Rp_	(	−3.6	;	12.2	;	32.6	)		
		A_0Pr_	(	0.5	;	6.9	;	92.3	)	A_0Pr_	(	18.1	;	0.8	;	22.0	)	A_0Pr_	(	−17.6	;	6.1	;	70.3	)		
		∑		0.7		∑		143.6		∑		26.3	∑	∑		35.8		∑		−25.6		∑		∑			
		f_cr_	(	0.2	;	26.2	;	2.6	)	f_cr_	(	0.6	;	28.1	;	10.0	)	f_cr_	(	−0.4	;	−1.9	;	−7.3	)		
		f_tb_	(	1.2	;	32.4	;	19.4	)	f_tb_	(	1.4	;	32.4	;	35.1	)	f_tb_	(	−0.2	;	0.0	;	−15.7	)		
		f_R_	(	3.2	;	26.2	;	75.4	)	f_R_	(	2.6	;	28.1	;	71.2	)	f_R_	(	0.6	;	−1.9	;	4.2	)		
		f_R_/2	(	8.5	;	13.1	;	175.8	)	f_R_/2	(	8.3	;	14.1	;	185.2	)	f_R_/2	(	0.2	;	−0.9	;	−9.4	)		
	BD2	A_0cr_	(	0.0	;	20.1	;	7.0	)	A_0cr_	(	1.2	;	2.6	;	1.2	)	A_0cr_	(	−1.2	;	17.4	;	5.8	)	0.3	3.5
		A_0M_	(	0.1	;	25.3	;	18.8	)	A_0M_	(	2.3	;	2.6	;	5.8	)	A_0M_	(	−2.3	;	22.6	;	13.0	)		
		A_0Rp_	(	0.1	;	20.1	;	51.8	)	A_0Rp_	(	12.9	;	1.6	;	29.1	)	A_0Rp_	(	−12.7	;	18.5	;	22.8	)		
		A_0Pr_	(	0.5	;	10.1	;	148.8	)	A_0Pr_	(	14.3	;	0.8	;	20.0	)	A_0Pr_	(	−13.8	;	9.2	;	128.8	)		
		∑		0.8		∑		226.4		∑		30.7	∑	∑		56.0		∑		−29.9		∑		∑			
		f_cr_	(	0.6	;	38.3	;	13.2	)	f_cr_	(	0.4	;	29.2	;	6.0	)	f_cr_	(	0.2	;	9.1	;	7.2	)		
		f_tb_	(	1.1	;	48.1	;	35.7	)	f_tb_	(	1.1	;	48.1	;	35.8	)	f_tb_	(	0.0	;	0.0	;	−0.1	)		
		f_R_	(	2.6	;	38.3	;	98.7	)	f_R_	(	5.2	;	29.2	;	186.3	)	f_R_	(	−2.6	;	9.1	;	−87.6	)		
		f_R_/2	(	9.3	;	19.1	;	283.3	)	f_R_/2	(	9.7	;	14.6	;	289.8	)	f_R_/2	(	−0.4	;	4.5	;	−6.5	)		
	BD3	A_0cr_	(	0.0	;	16.0	;	2.6	)	A_0cr_	(	0.7	;	1.8	;	0.4	)	A_0cr_	(	−0.7	;	14.2	;	2.2	)	0.2	2.0
		A_0M_	(	0.1	;	16.8	;	18.8	)	A_0M_	(	3.7	;	1.8	;	6.8	)	A_0M_	(	−3.6	;	15.0	;	12.0	)		
		A_0Rp_	(	0.1	;	16.0	;	38.4	)	A_0Rp_	(	32.8	;	1.5	;	52.7	)	A_0Rp_	(	−32.7	;	14.5	;	−14.4	)		
		A_0Pr_	(	0.9	;	8.0	;	198.2	)	A_0Pr_	(	27.1	;	0.7	;	25.2	)	A_0Pr_	(	−26.3	;	7.3	;	173.0	)		
		∑		1.1		∑		258.0		∑		64.3	∑	∑		85.1		∑		−63.2		∑		∑			
		f_cr_	(	0.4	;	30.5	;	4.9	)	f_cr_	(	0.2	;	26.9	;	2.1	)	f_cr_	(	0.4	;	3.6	;	2.9	)		
		f_tb_	(	1.4	;	31.9	;	35.8	)	f_tb_	(	1.4	;	31.9	;	37.2	)	f_tb_	(	−0.0	;	0.0	;	−1.5	)		
		f_R_	(	2.5	;	30.5	;	73.0	)	f_R_	(	11.7	;	26.9	;	310.2	)	f_R_	(	−9.2	;	3.6	;	−237.2	)		
		f_R_/2	(	15.6	;	15.2	;	377.3	)	f_R_/2	(	20.3	;	13.4	;	440.7	)	f_R_/2	(	−4.7	;	1.8	;	−63.4	)		
	BD4	A_0cr_	(	0.0	;	14.1	;	1.3	)	A_0cr_	(	1.0	;	1.6	;	0.9	)	A_0cr_	(	−1.0	;	12.5	;	0.4	)	0.2	1.9
		A_0M_	(	0.1	;	15.5	;	19.4	)	A_0M_	(	3.8	;	1.6	;	6.3	)	A_0M_	(	−3.8	;	13.9	;	13.0	)		
		A_0Rp_	(	0.2	;	14.1	;	39.5	)	A_0Rp_	(	2.4	;	1.6	;	4.2	)	A_0Rp_	(	−2.2	;	12.6	;	35.2	)		
		A_0Pr_	(	0.5	;	7.0	;	105.7	)	A_0Pr_	(	21.5	;	0.8	;	25.9	)	A_0Pr_	(	−20.9	;	6.3	;	79.8	)		
		∑		0.8		∑		165.8		∑		28.7	∑	∑		37.4		∑		−27.9		∑		∑			
		f_cr_	(	0.2	;	26.8	;	2.4	)	f_cr_	(	0.3	;	28.0	;	4.6	)	f_cr_	(	−0.1	;	−1.1	;	−2.2	)		
		f_tb_	(	1.5	;	29.6	;	36.9	)	f_tb_	(	1.5	;	29.6	;	37.4	)	f_tb_	(	−0.1	;	0.0	;	−0.5	)		
		f_R_	(	3.0	;	26.8	;	75.1	)	f_R_	(	2.3	;	28.0	;	59.2	)	f_R_	(	0.7	;	−1.1	;	15.9	)		
		f_R_/2	(	9.6	;	13.4	;	201.2	)	f_R_/2	(	9.0	;	14.0	;	193.4	)	f_R_/2	(	0.6	;	−0.6	;	7.8	)		
	BD5	A_0cr_	(	0.0	;	15.5	;	3.6	)	A_0cr_	(	1.2	;	2.1	;	1.3	)	A_0cr_	(	−1.2	;	13.5	;	2.3	)	0.2	2.5
		A_0M_	(	0.1	;	19.7	;	32.9	)	A_0M_	(	5.1	;	2.1	;	10.8	)	A_0M_	(	−5.0	;	17.6	;	22.1	)		
		A_0Rp_	(	0.2	;	15.5	;	72.3	)	A_0Rp_	(	7.7	;	1.6	;	15.3	)	A_0Rp_	(	−7.4	;	13.9	;	57.0	)		
		A_0Pr_	(	0.7	;	7.8	;	168.4	)	A_0Pr_	(	27.7	;	0.8	;	34.8	)	A_0Pr_	(	−27.0	;	7.0	;	133.6	)		
		∑		1.1		∑		277.2		∑		41.6	∑	∑		62.2		∑		−40.6		∑		∑			
		f_cr_	(	0.4	;	29.6	;	6.8	)	f_cr_	(	0.4	;	29.3	;	6.6	)	f_cr_	(	0.0	;	0.3	;	0.2	)		
		f_tb_	(	2.0	;	37.6	;	62.5	)	f_tb_	(	2.0	;	37.6	;	62.4	)	f_tb_	(	0.0	;	0.0	;	0.1	)		
		f_R_	(	4.3	;	29.6	;	137.7	)	f_R_	(	4.4	;	29.3	;	141.8	)	f_R_	(	−0.1	;	0.3	;	−4.2	)		
		f_R_/2	(	13.0	;	14.8	;	320.6	)	f_R_/2	(	13.1	;	14.6	;	322.1	)	f_R_/2	(	−0.1	;	0.2	;	−1.5	)		
	BD6	A_0cr_	(	0.0	;	10.3	;	1.5	)	A_0cr_	(	0.4	;	1.5	;	5.3	)	A_0cr_	(	−0.4	;	8.8	;	−3.9	)	0.1	1.8
		A_0M_	(	0.1	;	14.3	;	26.6	)	A_0M_	(	0.2	;	1.5	;	3.3	)	A_0M_	(	−0.1	;	12.8	;	23.2	)		
		A_0Rp_	(	0.2	;	10.3	;	59.1	)	A_0Rp_	(	0.0	;	1.1	;	0.6	)	A_0Rp_	(	0.2	;	9.2	;	58.6	)		
		A_0Pr_	(	0.8	;	5.2	;	126.2	)	A_0Pr_	(	15.7	;	0.6	;	19.0	)	A_0Pr_	(	−14.8	;	4.6	;	107.2	)		
		∑		1.2		∑		213.4		∑		16.3	∑	∑		28.2		∑		−15.1		∑		∑			
		f_cr_	(	0.3	;	19.7	;	2.8	)	f_cr_	(	0.1	;	20.4	;	27.5	)	f_cr_	(	0.1	;	−0.7	;	−24.7	)		
		f_tb_	(	1.9	;	27.3	;	50.6	)	f_tb_	(	0.2	;	27.2	;	44.7	)	f_tb_	(	1.8	;	0.0	;	5.8	)		
		f_R_	(	4.6	;	19.7	;	112.5	)	f_R_	(	0.2	;	20.4	;	47.6	)	f_R_	(	4.4	;	−0.7	;	64.9	)		
		f_R_/2	(	14.7	;	9.8	;	240.3	)	f_R_/2	(	5.1	;	10.2	;	146.0	)	f_R_/2	(	9.6	;	−0.3	;	94.3	)		
IV	concr. average	A_0_ = f_0_	(	0.4	;	34.4	;	6.9	)	A_0_ = f_0_	(	0.4	;	34.4	;	6.9	)	A_0_ = f_0_	(	0.0	;	0.0	;	0.0	)	0.1	x
	057	A_0cr_	(	0.0	;	6.0	;	0.4	)	A_0cr_	(	1.1	;	1.1	;	1.5	)	A_0cr_	(	−1.1	;	4.8	;	−1.1	)	0.1	1.1
		A_0M_	(	0.0	;	6.4	;	7.6	)	A_0M_	(	2.5	;	1.1	;	5.3	)	A_0M_	(	−2.4	;	5.3	;	2.2	)		
		A_0Rp_	(	0.1	;	6.0	;	12.5	)	A_0Rp_	(	1.3	;	1.1	;	2.8	)	A_0Rp_	(	−1.2	;	4.9	;	9.7	)		
		A_0Pr_	(	0.3	;	3.0	;	44.5	)	A_0Pr_	(	14.2	;	0.5	;	19.9	)	A_0Pr_	(	−13.9	;	2.5	;	24.6	)		
		∑		0.4		∑		65.0		∑		19.0	∑			29.5		∑		−18.6		∑		35.4	∑		
		f_cr_	(	0.1	;	34.9	;	2.4	)	f_cr_	(	0.4	;	36.2	;	10.4	)	f_cr_	(	−0.3	;	−1.3	;	−8.0	)		
		f_tb_	(	1.3	;	37.7	;	44.5	)	f_tb_	(	1.4	;	37.7	;	47.1	)	f_tb_	(	−0.1	;	0.0	;	−2.5	)		
		f_R_	(	2.1	;	34.9	;	73.3	)	f_R_	(	1.9	;	36.2	;	65.9	)	f_R_	(	0.1	;	−1.3	;	7.4	)		
		f_R_/2	(	9.6	;	17.5	;	261.3	)	f_R_/2	(	7.7	;	18.1	;	202.6	)	f_R_/2	(	1.9	;	−0.6	;	58.7	)		
	058	A_0cr_	(	0.0	;	6.8	;	1.1	)	A_0cr_	(	0.9	;	1.2	;	1.1	)	A_0cr_	(	−0.9	;	5.6	;	0.1	)	0.1	1.1
		A_0M_	(	0.1	;	6.9	;	19.6	)	A_0M_	(	6.7	;	1.2	;	15.8	)	A_0M_	(	−6.6	;	5.8	;	3.8	)		
		A_0Rp_	(	0.1	;	6.8	;	22.6	)	A_0Rp_	(	1.0	;	1.2	;	2.4	)	A_0Rp_	(	−0.9	;	5.7	;	20.2	)		
		A_0Pr_	(	0.2	;	3.4	;	42.5	)	A_0Pr_	(	9.8	;	0.6	;	17.3	)	A_0Pr_	(	−9.6	;	2.8	;	25.3	)		
		∑		0.4		∑		85.8		∑		18.4	∑	∑		36.5		∑		−18.0		∑		∑			
		f_cr_	(	0.3	;	40.1	;	6.6	)	f_cr_	(	0.3	;	40.1	;	7.3	)	f_cr_	(	−0.1	;	−0.0	;	−0.7	)		
		f_tb_	(	3.0	;	40.7	;	115.2	)	f_tb_	(	3.0	;	40.7	;	115.7	)	f_tb_	(	−0.1	;	0.0	;	−0.5	)		
		f_R_	(	3.4	;	40.1	;	132.5	)	f_R_	(	3.5	;	40.1	;	131.8	)	f_R_	(	−0.1	;	−0.0	;	0.7	)		
		f_R_/2	(	7.3	;	20.0	;	249.8	)	f_R_/2	(	7.4	;	20.1	;	250.2	)	f_R_/2	(	−0.1	;	−0.0	;	−0.3	)		
	059	A_0cr_	(	0.0	;	5.5	;	0.4	)	A_0cr_	(	0.6	;	1.0	;	0.9	)	A_0cr_	(	−0.6	;	4.5	;	−0.5	)	0.1	1.0
		A_0M_	(	0.0	;	5.9	;	2.4	)	A_0M_	(	0.6	;	1.0	;	1.1	)	A_0M_	(	−0.6	;	4.9	;	1.3	)		
		A_0Rp_	(	0.1	;	5.5	;	16.8	)	A_0Rp_	(	2.0	;	1.0	;	4.0	)	A_0Rp_	(	−1.9	;	4.6	;	12.8	)		
		A_0Pr_	(	0.2	;	2.8	;	32.1	)	A_0Pr_	(	13.9	;	0.5	;	21.4	)	A_0Pr_	(	−13.7	;	2.3	;	10.7	)		
		∑		0.3		∑		51.8		∑		17.1	∑	∑		27.4		∑		−16.7		∑		∑			
		f_cr_	(	0.1	;	32.5	;	2.5	)	f_cr_	(	0.3	;	33.0	;	6.3	)	f_cr_	(	−0.1	;	−0.5	;	−3.8	)		
		f_tb_	(	0.6	;	34.9	;	14.1	)	f_tb_	(	0.5	;	34.9	;	14.1	)	f_tb_	(	0.1	;	0.0	;	0.1	)		
		f_R_	(	3.1	;	32.4	;	98.7	)	f_R_	(	1.3	;	33.0	;	41.3	)	f_R_	(	1.8	;	−0.6	;	57.4	)		
		f_R_/2	(	7.0	;	16.2	;	188.8	)	f_R_/2	(	6.9	;	16.5	;	188.2	)	f_R_/2	(	0.1	;	−0.3	;	0.6	)		
